# Avoid or verify? How do social media users navigate cognitive conflict in health information on social media platforms?

**DOI:** 10.3389/fpubh.2025.1629777

**Published:** 2025-10-14

**Authors:** Chengyi Le, Zixin Wang, Jiahui Cheng, Ting Lu

**Affiliations:** ^1^School of Bussiness, Ningbo University, Ningbo, China; ^2^Merchants’ Guild Economics and Cultural Intelligent Computing Laboratory, Ningbo University, Ningbo, China; ^3^School of Economics and Management, East China Jiaotong University, Nanchang, China; ^4^Institute of China Non-Public Economy, Ningbo University, Ningbo, China; ^5^College of Education, Capital Normal University, Beijing, China

**Keywords:** social media, health information, cognitive conflict, information avoidance, information verification

## Abstract

Under the background of the digital intelligence era, users easily access diverse health information with varying perspectives through multiple social media channels, often falling into a dilemma of informational cognitive conflict. However, there is still a lack of systematic research on the internal mechanisms and boundary conditions that drive users to adopt different information behavior strategies under cognitive conflict. Based on cognitive dissonance theory, this study explores the influence of users’ cognitive conflict on different types of health information behavior and the underlying mechanisms. It further analyzes how health information with different characteristics can trigger information avoidance and information verification behaviors. In the first stage, a questionnaire survey was conducted and the hypotheses were tested using PLS-SEM. The results show that cognitive conflict positively influences users’ health information avoidance behavior through perceived information fatigue, but its effect on information verification behavior is not significant. In the second stage, experimental studies were conducted using different scenarios to further reveal the interaction effects of information relevance and information credibility on users’ health information behaviors. The results indicate that when both information relevance and credibility are high, users are more likely to engage in active information verification. In contrast, low relevance or low credibility tends to lead to information avoidance. Perceived information curiosity and perceived information fatigue play significant mediating roles in this process. This study expands the scope of research on users’ health information behaviors, deepens the understanding of cognitive dissonance theory in health information contexts, and provides theoretical support and practical guidance for the effective dissemination and utilization of health information. The research context may have certain limitations. Future studies could broaden sample sources and conduct empirical tests across different cultural contexts.

## 1 Introduction

With the rapid development of information technology, social media platforms have become deeply integrated into people’s daily lives, serving as important channels for accessing information and browsing news. In the wave of the digital intelligence era, the coverage and dissemination methods of health information have undergone unprecedented changes. The rise and widespread popularity of social media platforms have brought users a rich variety of health information services, enabling easier access, filtering, and utilization of health knowledge. However, while social media offers convenience in accessing health information, it also faces the challenge of uneven information quality. A large amount of distorted health information and health-related rumors circulate online, coexisting with high-quality health information and resulting in an overly complex information environment (1). Due to the diversity and complexity of information on social media, users often encounter inconsistencies or mismatches between the content they browse and their existing knowledge or attitudes, leading to cognitive conflict (2). This issue is particularly prominent in the domain of health information, where users often struggle to determine the authenticity of conflicting information, easily contributing to the emergence and spread of an “infodemic,” which poses a significant challenge to users’ information processing capabilities (3).

Although existing literature has provided abundant research on how cognitive conflict in health information influences users’ interactive behaviors, there remains a lack of systematic comparisons and analysis of the causes behind different types of interactive information behaviors under cognitive conflict (4, 5). Firstly, current research primarily focuses on how cognitive conflict influences low-interactivity behaviors such as information avoidance (6), whereas discussions of high-interactivity behaviors, such as information verification, are relatively limited. Moreover, few studies have compared the influences of cognitive conflict on different types of health information behaviors. Secondly, findings on the effects of cognitive conflict remain inconsistent: some scholars argue that it induces negative emotions in users, leading to health information avoidance, while others suggest it heightens users’ sense of uncertainty, prompting them to verify health information to reduce that uncertainty (7–9). These differing results may be attributable to variations in research contexts and methodologies. Lastly, although prior studies have examined the impact of cognitive conflict on users’ health information behavior, most have investigated either information avoidance or verification in isolation. Few studies have revealed the internal psychological mechanisms behind different information behaviors or explored the boundary conditions influencing such behaviors (5, 8).

Therefore, this study aims to address the following questions: When browsing health information on social media platforms, does cognitive conflict simultaneously influence users’ information verification and information avoidance behaviors? What are the internal psychological mechanisms that drive users to adopt different information behavior strategies under cognitive conflict? Do different features of health information (relevance and credibility) have varying effects on users’ tendencies to verify or avoid information? Through what mediating psychological mechanisms do these effects occur? To fill gaps in prior academic research, this study is based on cognitive dissonance theory and focuses on cognitive conflict in the context of health information on social media. Using a combination of questionnaire surveys and scenario-based experiments, it explores how cognitive conflict influences users’ health information verification and avoidance behaviors. The findings contribute to the existing body of knowledge on health information behaviors, broaden the research perspective on cognitive conflict, and offer practical guidance for improving health information dissemination strategies on social media platforms and enhancing public health literacy.

## 2 Literature review

### 2.1 Cognitive dissonance theory

Cognitive dissonance theory was first proposed by social psychologist Festinger. This theory reveals that when individuals are confronted with two or more conflicting cognitions, they experience psychological discomfort, a state referred to as cognitive conflict (10). People tend to adopt various strategies to alleviate this discomfort, which can be categorized into three main types: changing behavioral cognitive elements, adding new cognitions, and altering the relative importance of existing cognitions.

At present, cognitive dissonance theory has been widely studied and applied in fields such as psychology, management, and information science, mainly to explain the relationship between users’ information behaviors and their attitudes. For example, Jeong et al. based on cognitive dissonance theory, found that users of social networking services (SNS) experience psychological discomfort when exposed to differing viewpoints, and demonstrated that under such conditions, users are more likely to engage in selective exposure rather than emotional responses such as persuasion or rebuttal (11). Tsang’s research showed that encountering attitude-inconsistent information leads to belief conflict and negative emotions. Individuals experiencing dissonance tend to seek consistent information or avoid the situation altogether. More importantly, research has found that while cognitive discrepancy may trigger a desire to seek confirmatory information, only those who experience negative emotions are likely to adopt selective exposure as a strategy to reduce dissonance (12). Narayan et al. found that information avoidance is a common phenomenon in people’s daily lives, especially when information of personal concern may lead to cognitive dissonance. Such avoidance behaviors can be classified into passive and active forms (13).

Therefore, cognitive dissonance theory provides a valuable theoretical framework for understanding the various health information behaviors that arise following cognitive conflict. This dissonance represents a state of psychological imbalance experienced by users when facing conflicting cognitions. A deeper understanding and application of this theory can help us better analyze and predict users’ behaviors and decision-making processes in the context of information selection (14). This study introduces multiple variables to explore the internal psychological mechanisms and boundary conditions that lead to different types of information behaviors. By investigating the effects of cognitive dissonance in depth, we can enrich its application scenarios, better assist users in processing information and making decisions effectively, and reduce the occurrence of negative behaviors.

### 2.2 Information avoidance and information verification

Information avoidance and information verification are both important components of users’ information behavior. When processing health information, individuals may adopt five types of information behaviors—ranging from avoidance to verification—with increasing levels of interactivity, depending on their needs, attitudes, and external influences (15). Currently, there is no universally accepted definition of information avoidance in academia. Existing research generally regards information avoidance as an effective defensive mechanism, strategy, or response that individuals employ to cope with the cognitive or emotional discomfort caused by acquired or potential information (16). When users perceive certain information as unnecessary, they may consciously choose to ignore or avoid it due to limitations such as time, energy, knowledge, or personal interest (17, 18). In the context of health information, information verification behavior refers to users’ actions to verify the content of health information encountered on social media—based on the information itself or related cues—through methods such as online searches (8). With the rapid advancement of digital intelligence technologies and the growing public demand for information, research on information behavior has garnered wide attention in fields such as health, finance, and journalism. Health information behavior has become a prominent topic in the field of library and information science. In this paper, health information avoidance and health information verification are regarded as sub-branches of information behavior within the domain of health information.

Existing literature on users’ information behavior has primarily focused on adoption, seeking, and avoidance behaviors. For example, Soroya et al. found that during the process of acquiring information on social media, users may experience information overload and anxiety due to varying information sources, which in turn leads to information avoidance (19). Link investigated news consumers’ information avoidance during the COVID-19 pandemic, and the findings suggested that avoidance was associated with information-seeking attitudes, negative affective risk responses, descriptive and injunctive avoidance norms, and perceived information overload. Among these, attitudes and overload were the strongest predictors of avoidance behavior (7). Guo et al., using the stressor-strain-outcome framework, empirically examined how information irrelevance and overload contribute to social network fatigue and their relationship with users’ information avoidance behavior (20). In contrast, studies specifically focusing on information verification behavior are relatively scarce. Edgerly et al. explored participants’ intentions to verify information by manipulating headline veracity, source credibility, and alignment with users’ views (21).

In summary, most current studies have only analyzed information avoidance or verification behavior on social media in isolation and lack systematic investigation into the cognitive conflicts that drive these distinct types of information behavior. Therefore, this study introduces these two information behaviors into the field of health information behavior to explore the underlying psychological mechanisms that lead to different behaviors. It provides a new perspective for research on health information behavior and promotes the sustainable development of social media platforms.

## 3 Hypotheses

### 3.1 Cognitive conflict and users’ health information behavior

In the era of social media, due to the complexity of the online environment and the existence of information filtering, individuals are more likely to encounter health information that differs from their preexisting views, leading them into a state of cognitive conflict. Cognitive conflict, also known as cognitive inconsistency, refers to the incompatibility between one’s existing cognitive structure and new incoming cognition—this inconsistency of beliefs results in a state of cognitive dissonance (12). In the context of this study, cognitive conflict refers to the psychological tension or contradiction experienced by users when their preexisting cognitions are inconsistent with the knowledge or viewpoints embedded in the health information they encounter while browsing social media. According to cognitive dissonance theory, when individuals experience cognitive conflict, they may adopt various strategies to avoid dissonance, and such a dilemma may lead to a shift between information verification and information avoidance behaviors (10).

The academic community has conducted extensive research on the relationship between users’ cognitive conflict and their information behavior, though most studies have primarily focused on information avoidance. For example, Narayan et al. found that information avoidance is a common phenomenon in people’s everyday lives, especially when exposure to certain information may result in cognitive conflict. They identified two types of avoidance: passive and active (13). Golman et al. argued that individuals tend to avoid information that contradicts their existing beliefs in order to prevent such information from interfering with future decision-making (22). Dai et al. discovered that in the context of public health emergencies, due to the diversity of information sources, conflicting information from different channels often leads users to experience a series of complex psychological and cognitive changes during the information-seeking process—ultimately prompting a shift from seeking to avoidance behavior (4). Jeong et al., using covariance-based structural equation modeling, found that the more frequently users engaged with social media services, the more likely they were to encounter opposing views, which increased their discomfort. To alleviate this discomfort, users tended to adopt selective exposure strategies (11). On the contrary, some studies have confirmed that cognitive conflict can also lead to information verification behavior, which is the opposite of information avoidance. For instance, Huang et al., through interviews with 35 university students, found that cognitive conflict was a key driving factor for information verification behavior during online information acquisition (8).

These differing conclusions may stem from variations in research contexts and methodologies. In social media environments, cognitive conflict related to health information may trigger different psychological responses in users, leading to either information avoidance or verification behavior, thereby producing a dual-effect mechanism. The findings of this study have important theoretical and practical implications for understanding how cognitive conflict affects users’ information behavior in social media settings. In summary, this study posits that cognitive conflict experienced by users while browsing health information on social media induces psychological discomfort. This emotional state not only promotes information avoidance, leading users to steer clear of health information that might provoke further conflict, but also leads to information verification, motivating users to scrutinize the accuracy of the health information more carefully. Based on the above, this study proposes the following hypotheses:

*H*1a: Cognitive conflict positively influences users’ information verification behavior.

*H*1b: Cognitive conflict positively influences users’ information avoidance behavior.

### 3.2 The mediating role of perceived information curiosity and perceived information fatigue

Perceived information curiosity refers to a strong desire and motivation to seek and explore information triggered by external stimuli. This desire acts as an intrinsic driving force that motivates individuals to learn and acquire information (9). In the information age, the channels through which people access health information have become increasingly diverse and accessible. With the rapid expansion of information sources and platforms, the likelihood of encountering health information that conflicts with one’s existing cognition—whether through active search or passive exposure—has significantly increased (23). In fact, cognitive conflict often leads to heightened uncertainty, and this uncertainty can stimulate curiosity (9, 24). Perceived information curiosity helps us understand the relationship between cognitive conflict and information verification behavior in the context of social media health information. In situations involving cognitive conflict, curiosity can drive individuals to engage in health information verification behaviors in an effort to resolve inconsistencies and reduce uncertainty (8). Based on the above, this study proposes the following hypotheses:

*H*2a: Cognitive conflict positively influences users’ perceived information curiosity.

*H*3a: Perceived information curiosity positively influences users’ information verification behavior.

*H*4a: Perceived information curiosity mediates the relationship between cognitive conflict and information verification behavior.

As for perceived information fatigue, there is currently no universally accepted definition in the academic community. Zhang et al. defined social media fatigue as a negative emotional response to social media activities, such as feelings of exhaustion, boredom, and declining interest (25). Perceived information fatigue is a psychological concept referring to users’ negative emotional reactions toward health information encountered on social media. It is widely applied in the study of discontinuous usage behaviors and information avoidance (26). In such scenarios, users often encounter cognitively conflicting health information while using social media platforms, which may lead to psychological discomfort (27). Prolonged exposure to such conflict can result in anxiety, mental burden, and other negative emotions that manifest as fatigue. In the context of social media, fatigue has been identified as a major emotional factor predicting users’ reduced engagement, such as discontinuation, selective exposure, or switching behaviors. Perceived information fatigue is considered a key emotional factor influencing users’ intention to avoid information on social media platforms (4). Perceived information fatigue is viewed as a mediator in the relationship between cognitive factors and user behavior. This study aims to explore the mediating role of perceived information fatigue in the relationship between cognitive conflict and information avoidance behavior. Based on the above, this study proposes the following hypotheses:

*H*2b: Cognitive conflict positively influences users’ perceived information fatigue.

*H*3b: Perceived information fatigue positively influences users’ information avoidance behavior.

*H*4b: Perceived information fatigue mediates the relationship between cognitive conflict and information avoidance behavior.

### 3.3 The interaction between information relevance and information credibility

In the Information System Success Model, information relevance, as a critical dimension of information quality, has garnered significant academic attention. Within the context of social media, this study defines information relevance as the degree to which the content of information on social media platforms aligns with and meets users’ needs (28). Prior research has shown that when social media users encounter potentially false information, the higher the information relevance, the more likely they are to engage in fact-checking, and their emotional responses are more easily influenced by highly relevant content (29). Based on the SSO theoretical framework, Guo et al. analyzed data from 341 WeChat Moments users and found that irrelevant information directly leads to information avoidance behavior. Moreover, social media fatigue acts as a mediating factor—mediating the impact of overload on avoidance behavior (20). These findings suggest that the degree of information relevance is closely associated with different user information behaviors: when information is highly relevant, users are more likely to verify it, whereas low relevance may directly trigger information avoidance.

According to the cue utilization theory, users typically analyze a range of cues embedded in the information to evaluate its quality and guide decision-making behaviors (30). The environment in which users undertake their information journeys is constantly changing, and in today’s complex media landscape, users are flooded with uncertain information. To make decisions, they rely on multiple cues, among which information credibility plays a vital role (31). In this study, information credibility refers to the degree to which users perceive the topic and content of the information to be trustworthy. Information credibility has long been a central topic in user information behavior research and continues to draw scholarly community interest. For example, Edgerly et al. found that when participants perceived news headlines as truthful, they showed stronger intentions to verify the information—an intention shaped by the perceived consistency between the headline and their own beliefs (21). Similarly, Huang et al. discovered that when users detect contradictions or inaccuracies in the information, they begin to doubt its credibility and may initiate verification behaviors (8). Trang et al. argued that untrustworthy information is often avoided or ignored by audiences, and the credibility of advertising content significantly influences viewers’ attitudes and purchase intentions (32). Based on these findings, this study posits that in contexts of cognitive conflict—especially amid the rapid spread of ambiguous health information on social media—the credibility of such information becomes a key factor affecting users’ information behavior decisions.

In summary, this study proposes that the interaction effect of information relevance and credibility in cognitively conflicting health information on social media influences users’ verification behaviors. Based on the aforementioned hypotheses, we further infer that users’ perceived information curiosity and perceived information fatigue mediate the interaction effect. Accordingly, this study puts forward the following hypotheses:

*H*5a: The interaction between information relevance and information credibility influences users’ information verification behavior. When both information relevance and information credibility are high, users are more likely to engage in verification; however, when either information relevance or information credibility is low, verification behavior is unlikely to be triggered.

*H*5b: The different characteristics of information (information relevance and information credibility) influence information verification behavior through perceived information curiosity. When both information relevance and information credibility are high, they can influence perceived information curiosity, thereby promoting users’ information verification behavior.

*H*6a: The interaction between information relevance and information credibility influences users’ information avoidance behavior. When either information relevance or information credibility is low, users are more likely to engage in avoidance; when both information relevance and information credibility are high, avoidance behavior is unlikely to be triggered.

*H*6b: The different characteristics of information (information relevance and information credibility) influence information avoidance behavior through perceived information fatigue. When either information relevance or information credibility is low, they can increase perceived fatigue, thereby promoting users’ information avoidance behavior.

**Figure 1:**
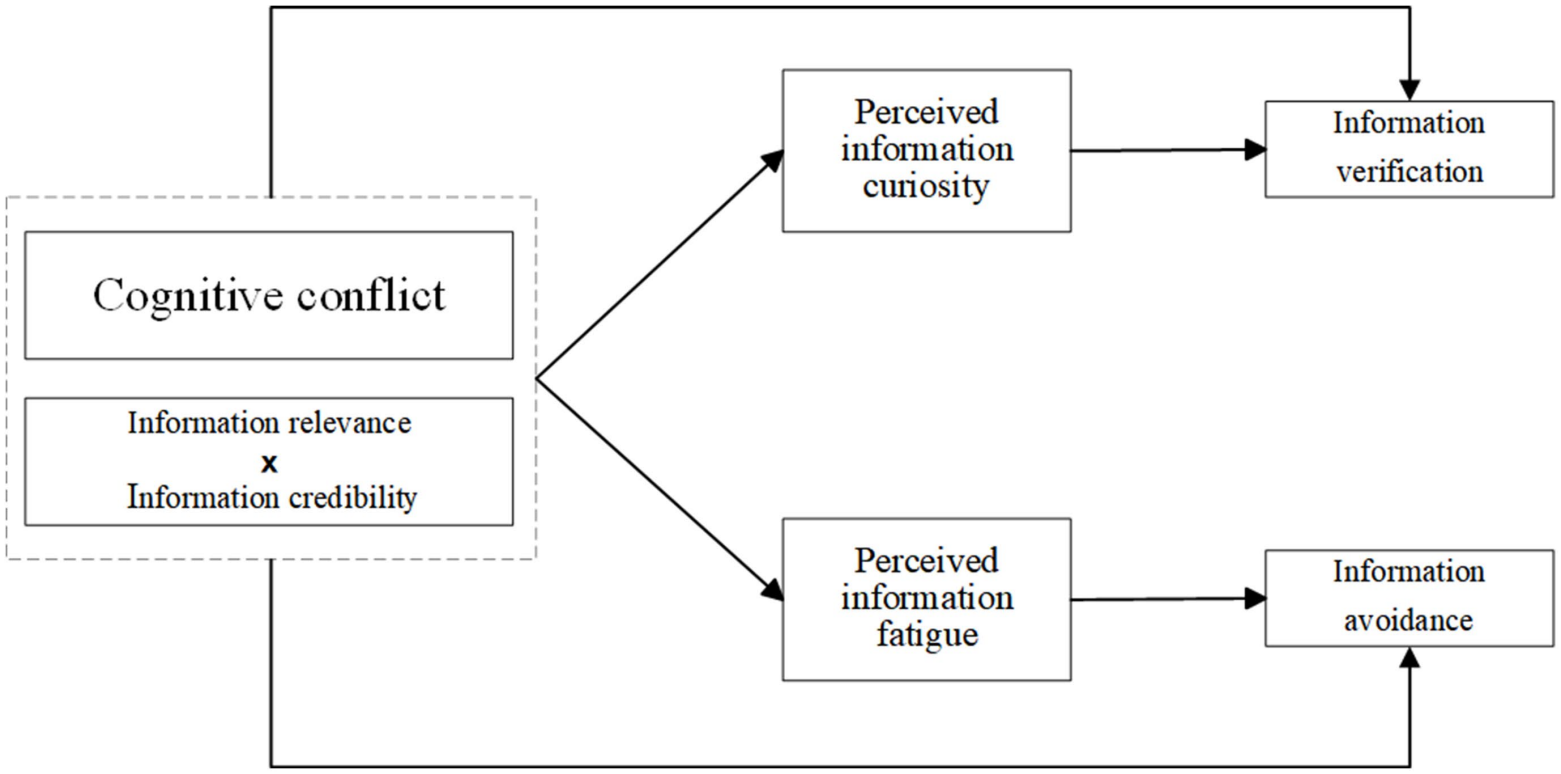
The theoretical model.

The research model is illustrated in Figure 1.

## 4 Study 1

### 4.1 Method

#### 4.1.1 Sample and data collection

This study employed a combination of offline paper-based questionnaires and online surveys for data collection. First, participants were recruited through multiple social media platforms such as zhihu, micro-blog, and rednote using the snowball sampling method, thereby covering social media users from diverse backgrounds. To further enhance sample diversity, the research team also conducted offline distribution of paper questionnaires using random sampling. In addition, online questionnaires were distributed via professional research platforms such as Credamo and Wenjuanxing, enabling access to a more diverse and heterogeneous pool of respondents nationwide. To ensure data integrity, the survey restricted each IP address to a single response. Participants were clearly informed that the survey was anonymous and strictly confidential, with all collected information used solely for academic research purposes. All respondents participated voluntarily and provided informed consent. A total of 286 participants were recruited for the survey. After eliminating invalid responses—such as those with excessively short completion times, inconsistent answers, or implausible responses—264 valid questionnaires were retained, resulting in an effective response rate of 92.31%. Among the respondents, 48.5% were male and 51.5% were female. The majority of participants (80.8%) were between the ages of 18 and 40, and 77.4% held a bachelor’s degree or higher, indicating a relatively broad and representative sample. Additionally, 68.18% of respondents reported spending more than 30 min per session on social media platforms, and 86.7% accessed health information via social media daily. The detailed sample characteristics are shown in Table 1. The demographic characteristics of the survey sample are consistent with the social media user statistics released by the China Internet Network Information Center (CNNIC), indicating that the data used in this study are not only suitable for further statistical analysis but also largely representative.

**Table 1:** Demographics data.

Variables	Characteristics	Count	Percentage
Gender	Male	128	48.5
	Female	136	51.5
Age group	Under 18	20	7.6
	18–25	129	48.9
	26–40	84	31.8
	41–55	20	7.6
	Over 55	11	4.1
Education qualification	High school and below	7	2.7
	Associate degree	51	19.3
	Bachelor’s degree	182	68.9
	Postgraduate degree and higher	24	9.1
Information acquisition frequency	Daily	229	86.7
	5–7 times a week	26	9.8
	2–4 times a week	8	3.0
	Once a week or less	1	0.3
Duration of each use	Less than 15 min	11	4.1
	15–30 min	73	27.7
	0.5–1 h	73	27.7
	1–2 h	71	26.9
	More than 2 h	36	13.6

#### 4.1.2 Instrument and measurement

The theoretical model of this study includes five variables. The measurement items for each latent variable were adapted from well-established scales developed in domestic and international research, ensuring content validity. All items were measured using a 7-point Likert scale, where “1” indicates “strongly disagree” and “7” indicates “strongly agree.” The measurement of cognitive conflict was based on the scale developed by Song et al. (33). Perceived information curiosity was measured using items from the scale designed by Agarwal and Karahanna (34). Perceived information fatigue was measured with reference to the scale developed by Zhang et al. (25). Information verification was measured using the scale created by Zha et al. (35), and information avoidance was assessed based on the scale developed by Dai et al. (4). To ensure the validity of the questionnaire items, a pre-test was conducted before the formal distribution. Ten graduate and doctoral students with relevant research backgrounds were invited to complete the questionnaire and provide feedback. Based on their suggestions, revisions were made to improve the questionnaire, resulting in the finalized version used in the formal study.

### 4.2 Measurement model

#### 4.2.1 Common method bias analysis

To avoid potential systematic errors caused by single-source data, this study implemented measures such as anonymous responses and screening criteria to control for common method bias. The Harman’s single-factor test was used to assess the impact of common method bias on the sample data. The results showed that, without any factor rotation, the first factor accounted for 26.55% of the total variance, which is below the 40% threshold. This indicates that there is no significant common method bias in this study.

#### 4.2.2 Reliability and validity

To verify the scientific validity and rationality of the research model, this study conducted both exploratory factor analysis (EFA) and confirmatory factor analysis (CFA) on the questionnaire data, with the results shown in Table 2. The Cronbach’s alpha values for all variables exceeded 0.7, and the composite reliability (CR) values ranged from 0.791 to 0.888, all above the 0.7 threshold, indicating good reliability and internal consistency of the theoretical model. The standardized factor loadings for the measurement items were generally above 0.7, and the average variance extracted (AVE) for each variable was higher than 0.5, demonstrating good convergent validity. Furthermore, the square roots of the AVE values for each variable were greater than the absolute values of the correlations between that variable and others, indicating good discriminant validity among the variables in this study.

**Table 2:** Reliability and validity analysis of the measurement model.

Construct	Items	Factor loading	Cronbach’s alpha	CR	AVE	CC	PIC	PIF	IV	IA
Cognitive Conflict	CC1	0.894	0.881	0.908	0.767	**0.876**				
	CC2	0.872								
	CC3	0.861								
Perceived Information Curiosity	PIC1	0.833	0.847	0.857	0.666	0.057	**0.816**			
	PIC2	0.814								
	PIC3	0.800								
Perceived Information Fatigue	PIF1	0.888	0.867	0.897	0.745	0.350	0.051	**0.863**		
	PIF2	0.861								
	PIF3	0.839								
Information Verification	IV1	0.863	0.895	0.895	0.740	0.088	0.651	0.022	**0.860**	
	IV2	0.862								
	IV3	0.856								
Information Avoidance	IA1	0.795	0.726	0.796	0.567	0.450	−0.291	0.506	−0.088	**0.753**
	IA2	0.788								
	IA3	0.669								

Correlations < diagonal AVE root, ensuring discriminant validity.

Boldface values on the diagonal indicate the square root of each construct’s AVE.

### 4.3 Structural model analysis

#### 4.3.1 Direct path analysis

This study uses Partial Least Squares Structural Equation Modeling (PLS-SEM) to test the theoretical framework and hypotheses. Currently, PLS-SEM is widely applied in the field of information resource management and is well-suited for exploring new conceptual models as well as analyzing higher-order constructs. The results of the hypothesis testing are shown in Table 3. In the context of social media, users’ cognitive conflict has a weak and non-significant effect on health information verification (*β* = 0.043, *p* = 0.429), thus H1a is not supported. However, cognitive conflict has a significant positive effect on health information avoidance (*β* = 0.318, *p* < 0.001), so H1b is supported. Cognitive conflict has a weak and non-significant effect on perceived information curiosity (*β* = 0.049, *p* = 0.446), meaning H2a is not supported. In contrast, it has a significant positive effect on perceived information fatigue (*β* = 0.318, *p* < 0.001), thus H2b is supported. During the user interaction process, both perceived information curiosity (*β* = 0.572, *p* < 0.001) and perceived information fatigue (*β* = 0.318, *p* < 0.001) have significant positive effects on information verification behavior and information avoidance behavior, respectively. Therefore, Hypotheses H3a and H3b are supported.

**Table 3:** Structural model.

Hypothesis	Path	Standard deviation (STDEV)	*p*-value
H1a	CC → IV	0.043	0.429
H1b	CC → IA	0.318	***
H2a	CC → PIC	0.049	0.446
H2b	CC → PIF	0.322	***
H3a	PIC→IV	0.572	***
H3b	PIF → IA	0.318	***

*p < 0.05, **p < 0.01, ***p < 0.001.

#### 4.3.2 Mediation analysis

To further examine the mediating effects of perceived information curiosity and perceived information fatigue, this study uses the Bootstrap method for analysis, with the results shown in Table 4. The indirect effect, total effect, and direct effect of cognitive conflict on health information verification behavior through perceived information curiosity are all non-significant. Perceived information curiosity does not mediate this relationship, so H4a is not supported. However, the indirect effect of cognitive conflict on user health information avoidance behavior through perceived information fatigue is significant (*β* = 0.134, *p* < 0.001), with the 95% confidence interval not containing 0. Additionally, the total effect (*β* = 0.304, *p* < 0.001) and direct effect (*β* = 0.170, *p* < 0.001) of this path are significantly positive, and the 95% confidence interval shown by Bootstrap also does not contain 0, indicating that the partial mediating effect of perceived information fatigue is significant. Therefore, H4b is supported.

**Table 4:** Indirect effects.

Hypothetical path	Effect	Effect size	Standard deviation	Bootstrap 95% CI	
				Lower	Upper
CC → PIC→IV	Total Effect	0.056	0.067	−0.076	0.189
	Direct Effect	0.016	0.055	−0.093	0.125
	Indirect Effect	0.040	0.040	−0.042	0.119
CC → PIF → IA	Total Effect	0.304	0.056	0.193	0.416
	Direct Effect	0.170	0.058	0.056	0.285
	Indirect Effect	0.134	0.031	0.077	0.200

In summary, study 1 uses a questionnaire survey method to study the relationship between cognitive conflict in health information on social media and users’ various information behaviors. The results show that cognitive conflict in health information positively influences users’ perceived information fatigue, which in turn promotes users’ health information avoidance behavior. However, the effect of cognitive conflict on perceived information curiosity and health information verification is not significant. Structural equation modeling is well-suited to handling the complex causal relationships in this study’s theoretical model, as it can reveal the underlying mechanisms among multiple variables and provide overall model validation. However, its limitation lies in its reliance on retrospective self-reported data, which makes it difficult to capture users’ dynamic psychological changes across different contexts. To address this limitation, Study 2 employed a scenario-based experimental method. By manipulating different experimental settings, it further tested the hypotheses and provided a more intuitive presentation of users’ reactions to diverse health information contexts. It should be noted that scenario experiments can effectively verify influence relationships between variables, but they are relatively limited in revealing the strength of these relationships, a gap that Study 1 with SEM helps to fill. The combination of these two methods not only validates the theoretical model at the overall path level but also provides experimental support in specific contexts, thereby achieving methodological complementarity. This design enhances the credibility of the inferred relationships, incorporates multiple standards of data validation, and simultaneously meets the requirements of manipulation checks as well as reliability and validity assessments, thus improving the reliability and robustness of the study’s conclusions.

## 5 Study 2

To verify whether different informational characteristics of health information under cognitive conflict lead to two distinct types of information behavior—information verification and information avoidance—and to examine the underlying psychological mechanisms identified in study 1, this study adopted a 2 (information relevance: high vs. low) × 2 (information credibility: high vs. low) between-subjects experimental design within the context of cognitive conflict. An online scenario-based experiment was conducted through a professional data research platform to further explore the interaction effects of information relevance and information credibility on users’ different health information behaviors in a cognitively conflicting context.

### 5.1 Pre-test

The purpose of the pre-test was to determine the manipulation materials for health information involving cognitive conflict. Focusing on health information shared on social media platforms, this study designed two types of experimental materials: health information with cognitive conflict and health information without cognitive conflict. For the design of materials involving cognitive conflict, a series of health information items was selected from authoritative platforms such as the China Internet Joint Rumor Debunking Platform^1^ and the official Weibo account of the China Association for Science and Technology’s Science Rumor Debunking Platform.^2^ All selected health information items were reviewed one by one, and highly circulated content was excluded. In the end, 20 health information items with cognitive conflict and 20 without cognitive conflict were retained. To ensure the scientific rigor of the survey, this study randomly recruited 12 scholars in the field of Library, Information, and Information Resources Management to conduct a pretest of the questionnaire. A total of 120 participants were recruited both offline and online to take part in the study and were compensated with cash red envelopes. The researchers informed the participants that the survey was about user health behavior on social media and distributed the questionnaire accordingly. Participants were randomly assigned to either the cognitive conflict group or the non-cognitive conflict group. The questionnaire presented 20 distinct pieces of health information, and participants were asked to rate the level of cognitive conflict perceived in each item. The measurement of cognitive conflict was based on a 7-point Likert scale adapted from Song et al. (33), including three items such as “This health information does not align well with my existing health knowledge and understanding.” Participants whose average cognitive conflict score was greater than 4 were categorized into the cognitive conflict group, while those scoring less than 4 were placed in the non-cognitive conflict group. An independent samples t-test showed that the cognitive conflict group scored significantly higher than the non-cognitive conflict group (M_cognitive conflict_ = 5.322, M_non-cognitive conflict_ = 2.067, *F*(1,120) = 3.496, *p* < 0.001). Therefore, the manipulation of cognitive conflict in these health information materials was deemed successful.

### 5.2 Method

The experimental materials for this study were sourced from social media platforms and a 2 (information relevance: high vs. low) × 2 (information credibility: high vs. low) between-subjects design was used. To ensure the experimental materials had discriminative power, eight participants (including doctoral and master’s students in library and information science, who did not participate in the formal survey) were invited before the official survey to categorize the pre-randomized materials based on the levels of information relevance and credibility. Preliminary modifications were made based on their feedback. Participants with an information relevance score greater than 4 were categorized into the high information relevance group, while those with a score less than 4 were categorized into the low information relevance group. The same categorization method was applied to information credibility. Sample materials are shown in Table 5. In the contextual experiment, this study again invited the participants from the first phase to conduct a follow-up survey, with 260 participants involved in this experiment. To avoid bias in the experimental results, this study used a double-blind randomized controlled experimental method, where participants were unaware of their group allocation until the experiment was over. Participants were randomly divided into four groups, with each group including three pieces of health information that differed in terms of information relevance and credibility. To improve the quality of the sample data, we conducted an audit on the data quality of the participants. After eliminating invalid samples such as those with too short completion times, contradictions, and excessive neutral responses, 237 valid sample data were obtained. Among them, there were 108 male participants (45.6%) and 129 female participants (54.4%). Regarding age distribution, participants were mostly between 18 and 40 years old, accounting for 84.8% of the total sample. 82.7% of the participants had at least a bachelor’s degree, indicating a relatively diverse sample. Additionally, there were no significant differences in gender, age, education level, or occupation across the four experimental groups, which ensures that the sample met the data analysis requirements.

**Table 5:** Examples of experimental materials with high/low information relevance and high/low information credibility.

Group	Experimental materials
1	Sugar oranges, yogurt and milk should not be eaten together.
2	Diabetic patients need to eat less staple food.
3	Regularly wearing black clothes may easily lead to cancer.
4	Regular oxygen inhalation can help you grow taller and improve your intelligence level.

The formal questionnaire consisted of four parts. The first part provided a brief introduction to the purpose of the survey and the instructions for completing it. We informed the participants that this experiment was about health information behavior and that the survey was anonymous, with the collected data being used for academic research only, ensuring the confidentiality of all survey content. The second part guided participants into different contexts of health information with varying levels of relevance and credibility through stimulus materials. In the third part, after reading the experimental materials, participants were required to complete measurement scales for perceived information curiosity (Cronbach’s *α* = 0.927), perceived information fatigue (Cronbach’s α = 0.950), information verification (Cronbach’s α = 0.943), and information avoidance (Cronbach’s α = 0.968), based on their feelings during the reading process. The measurement items for each variable were adapted from existing mature scales in domestic and international studies, with reasonable modifications based on the actual conditions of domestic users and the research theme to ensure the validity of the measurements. Perceived information curiosity was measured based on the scale designed by Agarwal and Karahanna (34), perceived information fatigue was measured based on the scale designed by Zhang et al. (25), information verification was measured based on the scale developed by Zha et al. (35), information avoidance was measured based on the scale designed by Dai et al. (4), information relevance was measured based on the scale developed by Kim et al. (36), and information credibility was measured based on the scale developed by Gao et al. (37), with appropriate revisions for the social media platform context. All variables were measured using a 7-point Likert scale, with 1 meaning “strongly disagree” and 7 meaning “strongly agree.” The final part consisted of demographic questions, where participants were asked to provide information about their gender, age, education level, and occupation.

### 5.3 Results

#### 5.3.1 Manipulation check

First, the effectiveness of the manipulation of information relevance was tested. The results indicated that the manipulation was successful. There was a significant difference in participants’ perceptions between the high and low information relevance conditions (M_high relevance_ = 5.523, SD = 0.754; M_low relevance_ = 2.762, SD = 0.668; *F*(1, 237) = 5.390, *p* < 0.001). Second, the manipulation of information credibility was tested. The results showed a significant difference in participants’ perceptions between the high and low information credibility conditions (M_high credibility_ = 5.409, SD = 0.792; M_low credibility_ = 2.207, SD = 0.831; F(1, 237) = 1.240, *p* < 0.001). Therefore, the manipulation of information credibility was successful.

#### 5.3.2 Hypothesis testing

**Figure 2:**
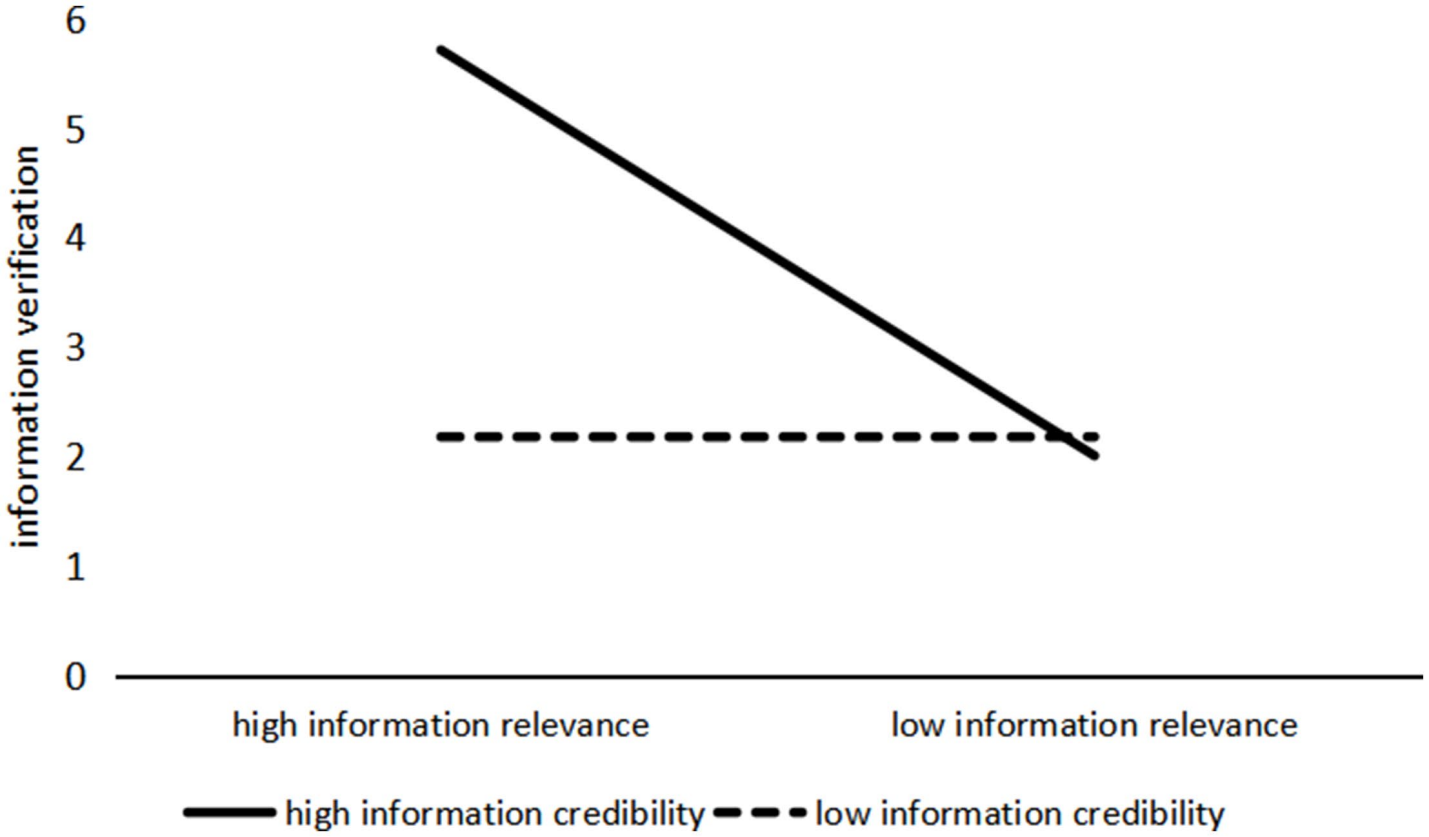
The interaction effect of information relevance and information credibility on information verification.

The ANOVA results with information verification as the dependent variable indicate a significant interaction effect between information relevance and information credibility (*F*(1, 237) = 494.796, *p* < 0.001). The interaction effect is illustrated in Figure 2. A further simple effects analysis revealed that when health information on social media had high information relevance, users exhibited significantly more information verification behavior toward high-credibility information than low-credibility information (M_high credibility_ = 5.714, SD = 0.080; M_low credibility_ = 2.179, SD = 0.087; *F*(1, 237) = 891.368, *p* < 0.001). However, when the health information had low information relevance, there was no significant difference in information verification behavior between the high and low credibility conditions (M_high credibility_ = 2.006, SD = 0.083; M_low credibility_ = 2.181, SD = 0.083; *F*(1, 237) = 2.225, *p* = 0.137). Therefore, H5a is supported.

**Figure 3:**
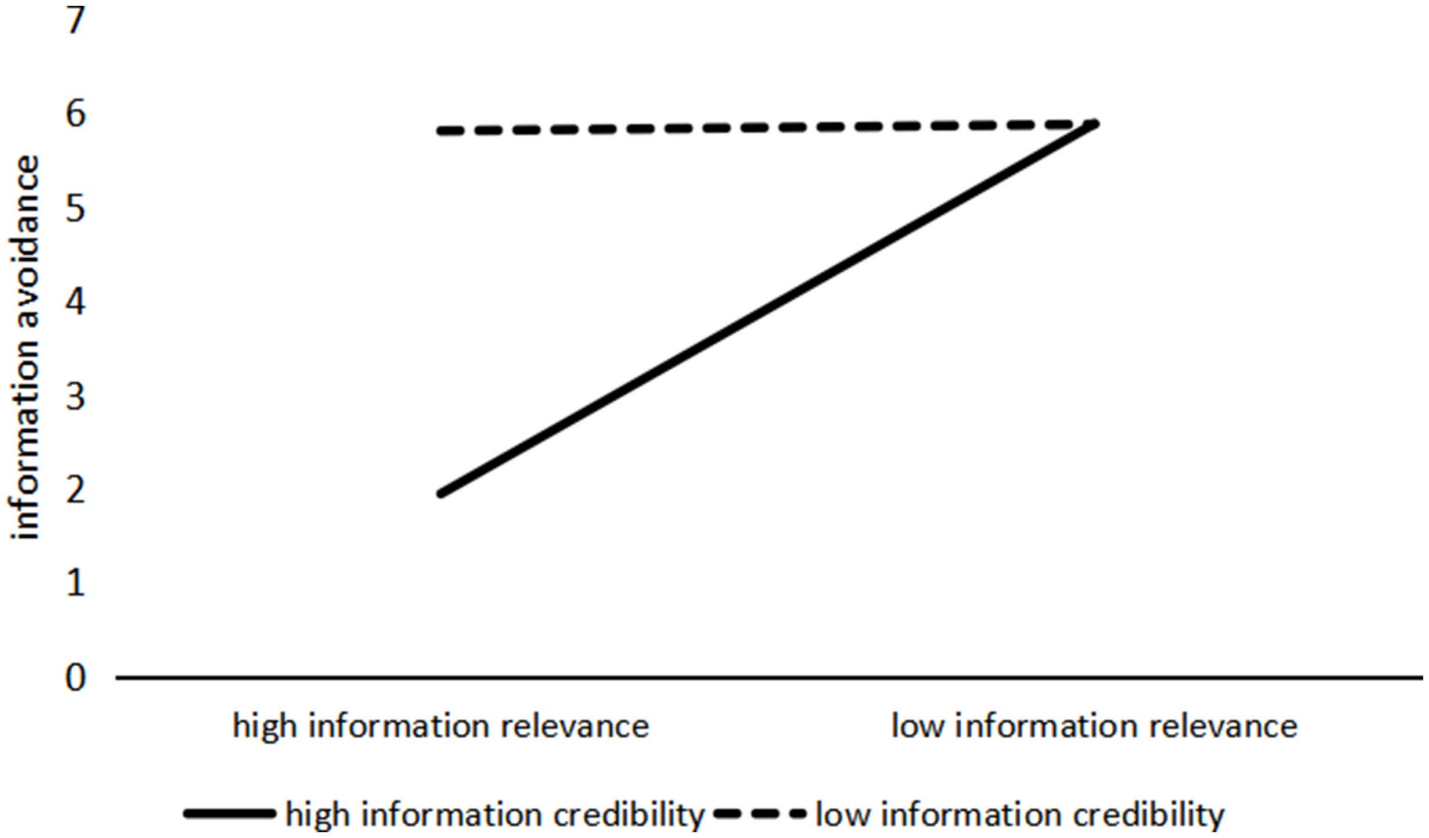
The interaction effect of information relevance and information credibility on information avoidance.

The ANOVA results with information avoidance as the dependent variable also reveal a significant interaction effect between information relevance and information credibility (*F*(1, 237) = 525.590, *p* < 0.001), as shown in Figure 3. A further simple effects analysis showed that when health information on social media had high information relevance, low-credibility information triggered significantly more information avoidance behavior than high-credibility information (M_high credibility_ = 1.943, SD = 0.081; M_low credibility_ = 5.883, SD = 0.088; *F*(1, 237) = 1081.215, *p* < 0.001). However, when the information had low relevance, there was no significant difference in information avoidance behavior between high and low credibility conditions (M_high credibility_ = 5.806, SD = 0.084; M_low credibility_ = 5.876, SD = 0.084; *F*(1, 237) = 0.348, *p* = 0.556). Therefore, H6a is supported.

#### 5.3.3 Mediation analysis

**Figure 4:**
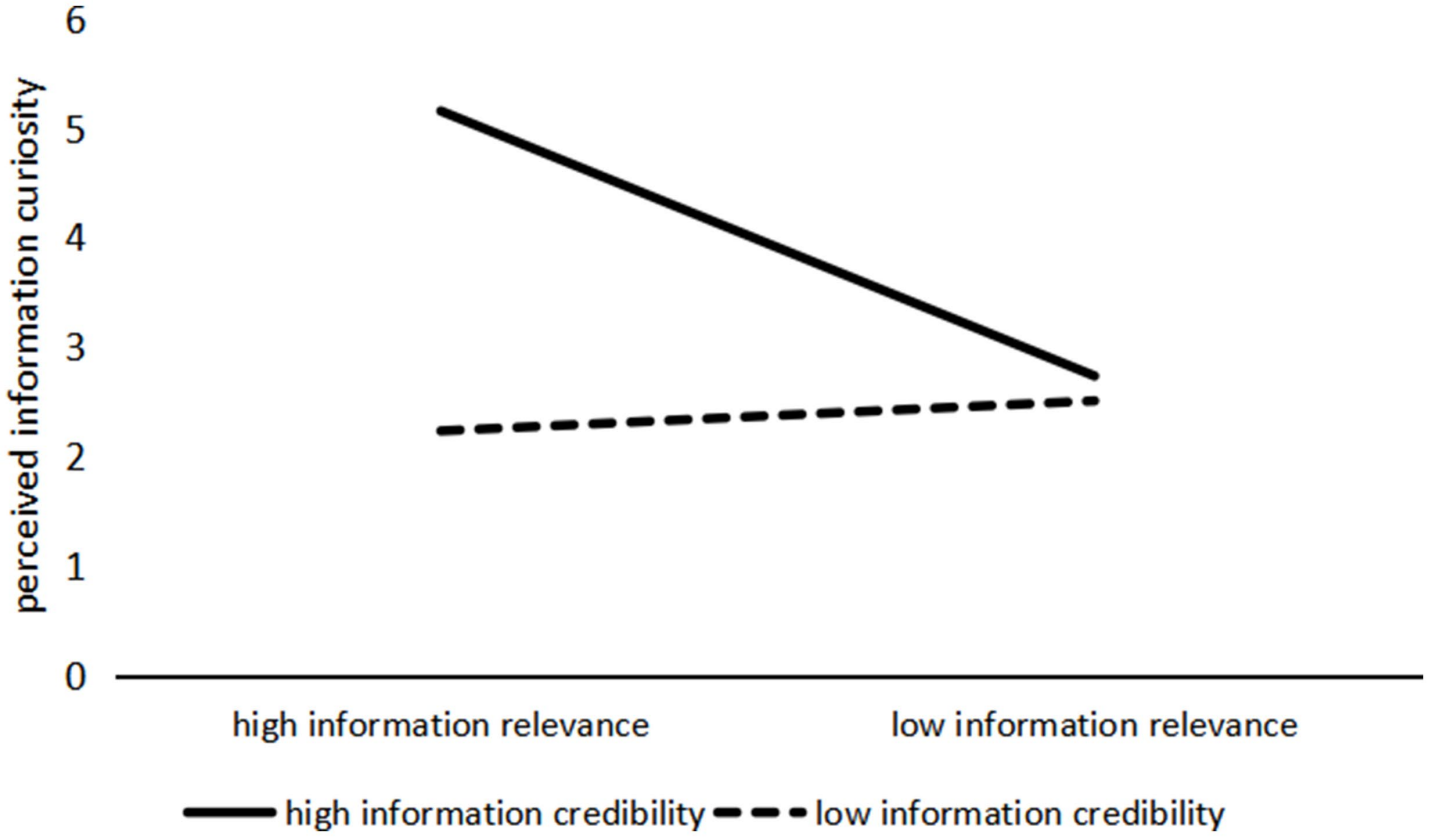
The interaction effect of information relevance and information credibility on perceived information curiosity.

**Figure 5:**
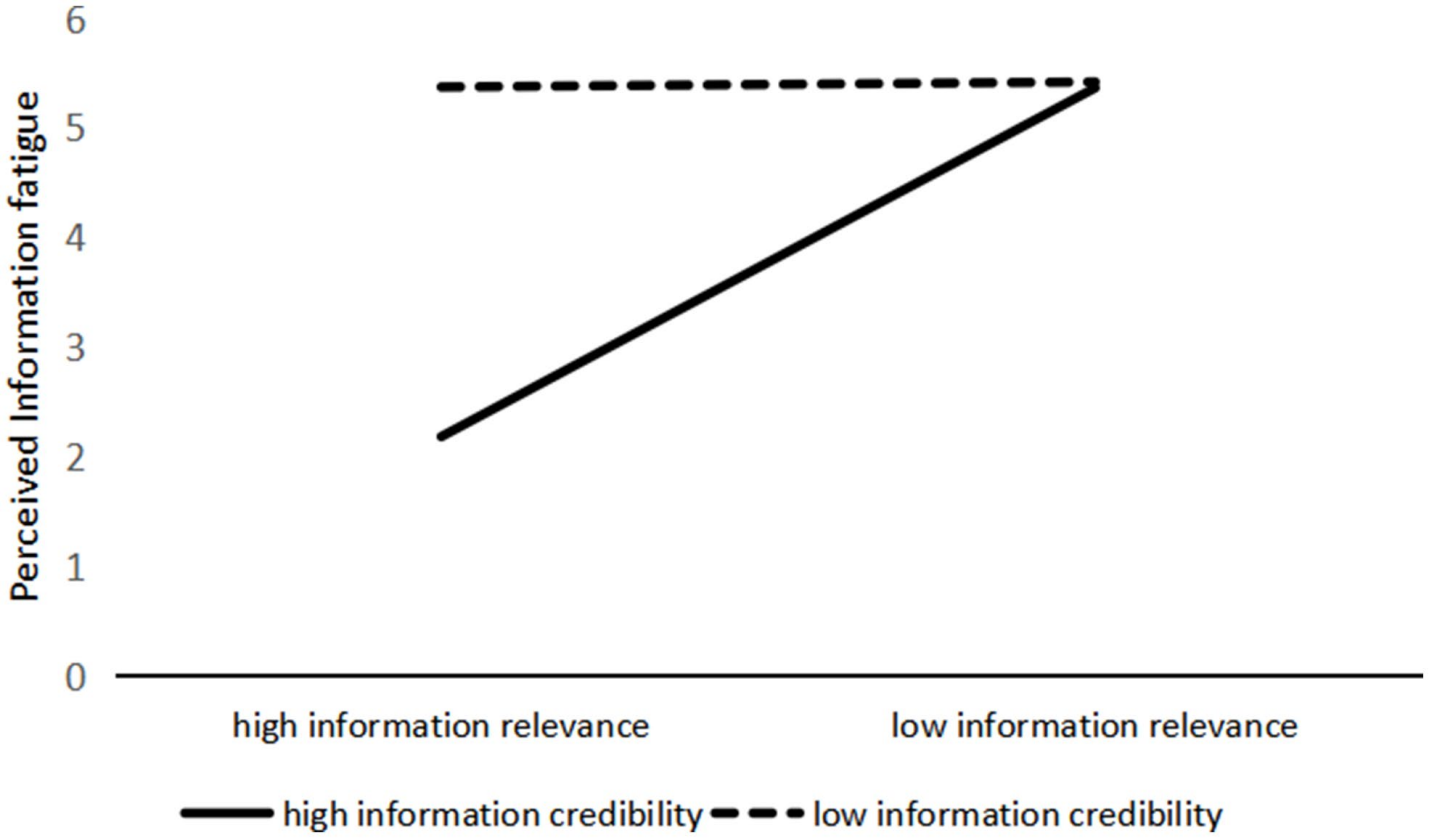
The interaction effect of information relevance and information credibility on perceived information fatigue.

First, the interaction effect of information relevance and information credibility on perceived information curiosity was tested. The ANOVA results (see Figure 4) showed a significant interaction effect (*F*(1, 237) = 161.461, *p* < 0.001). Specifically, when the information relevance of health information was high, participants exhibited significantly higher perceived information curiosity in response to high-credibility information compared to low-credibility information (M_high credibility_ = 5.156, SD = 0.102; M_low credibility_ = 2.735, SD = 0.111; *F*(1, 237) = 258.380, *p* < 0.001). However, when the information relevance was low, there was no significant difference in perceived information curiosity between the high- and low-credibility conditions (M_high credibility_ = 2.233, SD = 0.105; M_low credibility_ = 2.508, SD = 0.106; *F*(1, 237) = 3.388, *p* = 0.067). Second, the interaction effect of information relevance and information credibility on perceived information fatigue was also significant (F(1, 237) = 269.405, *p* < 0.001). When health information had high relevance, participants reported significantly higher perceived information fatigue in response to low-credibility information compared to high-credibility information (M_high credibility_ = 2.182, SD = 0.092; M_low credibility_ = 5.364, SD = 0.100; F(1, 237) = 550.397, *p* < 0.001). In contrast, when the information relevance was low, there was no significant difference in perceived information fatigue between high and low credibility (M_high credibility_ = 5.372, SD = 0.095; M_low credibility_ = 5.418, SD = 0.096; F(1, 237) = 0.116, *p* = 0.734), as shown in Figure 5.

Using Model 7 of the PROCESS macro, with 5,000 bootstrap samples and a 95% confidence interval, we tested the mediating effects of perceived information curiosity and perceived information fatigue on information verification as the dependent variable. The results showed that perceived information curiosity mediates the interactive effect of information relevance and information credibility on information verification behavior (LLCI = 2.030, ULCI = 2.893, not including 0). Further analysis of the mediating effect under different levels of information relevance revealed that in the high information relevance group, the mediating effect of perceived information curiosity was significant (LLCI = 2.181, ULCI = 3.178, not including 0), whereas in the low information relevance group, the mediating effect was not significant (LLCI = −0.348, ULCI = 0.219, including 0). These findings indicate that for health information with high relevance, perceived information curiosity mediates the effect of information relevance on information verification, whereas for low relevance, perceived information curiosity does not serve as a mediator. Thus, H5b is supported. The mediation analysis of perceived information fatigue in the interaction between information relevance and information credibility on information avoidance behavior also indicated a significant mediating effect (LLCI = −3.555, ULCI = −2.775, not including 0). In the high information relevance group, the mediating effect of perceived information fatigue was significant (LLCI = −3.450, ULCI = −2.930, not including 0), while in the low information relevance group, the mediating effect was not significant (LLCI = −0.326, ULCI = 0.218, including 0). These results suggest that for highly relevant health information, perceived information fatigue mediates the effect of information relevance on information avoidance behavior, whereas for low relevance, perceived information fatigue does not play a mediating role. Therefore, H6b is supported.

## 6 Discussions and implications

### 6.1 Findings and discussion

Based on cognitive dissonance theory, this study explored the impact of cognitive conflict in health information on different types of information behavior by users in social media contexts, and investigated the interaction effects of information relevance and credibility in the context of cognitive conflict on various health information behaviors. The theoretical model and research hypotheses were tested using two research methods, and the following conclusions were drawn:

First, this study verified the relationship between cognitive conflict and users’ health information behavior through a questionnaire survey. The findings indicate that users’ cognitive conflict regarding health information positively influences their health information avoidance behavior. This result is consistent not only with empirical studies conducted in the United States (38), but also with prior research in China (17). However, cognitive conflict does not have a significant effect on perceived information curiosity or health information verification behavior.

Second, users’ cognition and behavior in the information journey are influenced by emotional factors. Perceived information fatigue partially mediated the effect of cognitive conflict on information avoidance, while the mediating effect of perceived information curiosity was not significant, though it positively influenced information verification behavior.

Third, the study further explored the interaction effects of different levels of information relevance and credibility on users’ health information behaviors through a scenario-based experiment. The results indicated that when both information relevance and credibility are high, users are more likely to engage in active information verification. Conversely, low information relevance or low information credibility tends to lead to information avoidance. Both perceived information curiosity and perceived information fatigue played significant mediating roles in these effects.

### 6.2 Theoretical implications and practical implications

This study makes several key theoretical contributions:

First, this study further broadens the research field concerning the relationship between cognitive conflict and users’ health information behaviors. Although there has been a certain accumulation of research on health information behaviors, most existing studies have primarily focused on a single aspect—either users’ information verification behavior or information avoidance behavior—without fully exploring the impact of these two fundamentally different types of information behavior within the context of social media (29). By adopting a mixed-method approach that combines questionnaire surveys and scenario-based experiments, this study empirically demonstrates that cognitive conflict can simultaneously trigger both information verification and information avoidance behaviors, which differ in their interactive nature. Based on cognitive dissonance theory, this study explores its significant influence on users’ health information verification and avoidance behaviors. This not only represents an innovative exploration of users’ health information behaviors in the context of social media but also enriches the literature related to cognitive dissonance theory and user information behavior.

Second, this study reveals the underlying psychological mechanisms through which cognitive conflict in social media influences different information behaviors and provides new mediating variables for research on the effects of cognitive conflict. Existing literature has mostly identified psychological factors such as anxiety, trust, and perceived risk as key influences on users’ information behaviors (3). This study introduces perceived information curiosity and perceived information fatigue as mediating factors between cognitive conflict and different types of information behavior. Through two research methods, their mediating roles are validated. These findings contribute to uncovering the psychological mechanisms behind the cognitive conflict effect, offering a possible explanation for how cognitive conflict can lead to two divergent health information behaviors, and expanding the research perspective in this area.

Third, in the context of cognitive conflict, this study investigates, from the perspective of information characteristics, how information relevance and information credibility affect users’ different health information behaviors and their underlying mechanisms. Traditional questionnaire-based research often fails to fully capture users’ psychological changes across different contexts (18). This study draws on cue utilization theory and the Information Systems Success Model and employs scenario-based experimental methods to provide evidence for two different types of health information behavior under the same cognitive conflict scenario. It explains the psychological mechanism through which the interaction of information relevance and credibility influences user behavior, offering a possible explanation for the coexistence of contrasting information behaviors under cognitive conflict. These findings contribute novel insights to the research on user information interaction behaviors.

This study also provides the following practical implications for relevant organizations of social media platforms:

First, the findings show that cognitive conflict regarding health information is common among users of social media platforms, which calls for practical and effective measures. On one hand, platforms should provide effective information filtering mechanisms and strictly regulate health information that is likely to trigger users’ cognitive conflict, ensuring the authenticity and accuracy of content. On the other hand, they should improve the quality of health-related content and promote correct and useful health knowledge to ensure users’ access to high-quality information.

Second, relevant internet regulatory authorities should establish clear standards and strengthen oversight of the sources of health information. For health information on social media that fails to meet standards and is likely to cause cognitive conflict, timely governance actions should be taken to curb the spread of health-related misinformation. Governments, professional organizations, and social media platforms can collaborate to establish dedicated sections for health information governance, aiming to reduce the circulation of low-quality and misleading content. This would help lower the incidence of problems arising from cognitive conflict and provide users with a more reliable and accurate health information environment.

Last, relevant institutions should actively foster and enhance users’ health information literacy and promote interaction and communication between authoritative experts and the general public on social media. By using social media platforms and other widely accessible channels, broad campaigns can be conducted to promote health information literacy. This will help ordinary users improve their understanding of health knowledge and enhance their ability to identify cognitively conflicting or misleading health information. Such efforts will not only strengthen public belief in sound health knowledge but also effectively raise overall health literacy, laying a solid foundation for building a healthy and harmonious social environment.

### 6.3 Limitations and future research

This study still has certain limitations. First, the sample data has inherent constraints. Although data was collected through a combination of online and offline anonymous surveys across multiple channels, self-reported data may deviate from actual behavior. Future research could consider using web scraping or other methods to collect real-world secondary data to further validate the findings of this study. Second, the research focuses on user information behavior within the context of social media platforms. Given that user behavior and usage habits vary significantly across different environments, the contextual scope of this study may be limited. Future studies could further examine how cognitive conflict influences users’ health information behaviors in other settings and empirically test the proposed model across different cultural contexts to validate the cross-cultural robustness of our conclusions. Finally, while this study verifies the interaction effect between information relevance and information credibility—offering both theoretical and practical value—it should be noted that users’ information journeys are complex and dynamic. These journeys involve varying levels of interactivity and diverse information behaviors. Future research could investigate additional influencing factors, such as emotional arousal or health information literacy, to provide a more comprehensive understanding of user behavior.

## Data Availability

The original contributions presented in the study are included in the article/supplementary material, further inquiries can be directed to the corresponding author.

## Footnotes

^1^ http://www.piyao.org.cn/

^2^ http://weibo.com/u/6507165034
